# Risk Factors Predicting Complications of Transvenous Lead Extraction

**DOI:** 10.1155/2018/8796704

**Published:** 2018-12-18

**Authors:** Wojciech Jacheć, Anna Polewczyk, Maciej Polewczyk, Andrzej Tomasik, Marianna Janion, Andrzej Kutarski

**Affiliations:** ^1^2nd Department of Cardiology, Medical University of Silesia in Katowice, School of Medicine with the Division of Dentistry in Zabrze, Poland; ^2^Faculty of Medicine and Health Sciences, The Jan Kochanowski University, Kielce, Poland; ^3^2nd Department of Cardiology, Swietokrzyskie Cardiology Center, Kielce, Poland; ^4^Acute Cardiac Care Unit, Swietokrzyskie Cardiology Center, Kielce, Poland; ^5^Department of Cardiology, Medical University, Lublin, Poland

## Abstract

**Objective:**

Transvenous lead extraction (TLE) is the gold standard in the management of patients with cardiac implantable electronic devices (CIED)-related complications. Knowledge of TLE risk factors is very important.

**Methods:**

Clinical data from 1915 patients undergoing TLE at the Reference Center between 2006 and 2015 were analyzed. The effects of clinical and procedure-related factors on the development of major (MJC) and minor (MIC) complications and survival after TLE were evaluated.

**Results:**

MJC were caused mainly by lead implant duration, presence of abandoned leads, multiple procedures preceding TLE, and any technical problem during TLE. Of clinical factors female gender and anemia increased the risk of MJC. MIC were reported in patients with the first implantation of CIED under the age of 30 and after sternotomy analysis of 30-day survival after procedure demonstrated a significant effect of clinical factors and lead dwell times, previous unsuccessful TLE, and MIC.

**Conclusions:**

Efficacy and safety of TLE depend mainly on procedure-related factors. This knowledge is essential for preventing MJC and MIC. Mortality at 30 days following TLE is mainly associated with the clinical factors; however, there was also a significant effect of lead dwell time and periprocedural complications on the short-term prognosis of patients undergoing TLE.

## 1. Introduction

As cardiac implantable electronic devices (CIED) are increasingly widely used to treat patients with sinus node disease and atrioventricular block, to prevent sudden cardiac death and to manage patients with advanced heart failure, the incidence of pacemaker complications has been rising since the end of the 20^th^ century. Transvenous lead extraction (TLE) is the gold standard in the treatment of patients with CIED-related complications. Initial experiences in successful extraction of the leads by continuous traction were described in 1980 [1]. Countertraction, another intravascular technique for extraction of pacemaker leads in the early 1990s, together with the outer sheaths was used to disrupt fibrotic attachments and remove the lead. However, without diagonal cuts tremendous force was required, which prompted further investigations. In the mid-1990s, the Byrd telescoping dilator sheaths (Cook®, Pennsylvania) became available, being now one of the fundamental tools for dilating scar tissue and extracting the leads [2, 3]. In the next stage, a locking stylet was introduced to reduce the probability of stretching and breaking the lead during TLE [4].

Since the beginning of the 21^st^ century, the medical world has been witnessing further many improvements in transvenous lead removal including hand-powered evolution mechanical dilator sheaths Evolution (Cook®), TightRail (Spectranetics®, CA) rotating mechanical dilator sheaths as well as sheaths powered by ablative energy sources, i.e., excimer laser sheaths (SLS II Laser Sheath) or electrosurgical dissection sheaths (Spectranetics®), Perfecta (Cook®) [5–7]. The new tools, especially those using laser or electrical energy, are less time consuming and slightly more efficient; however they are probably associated with a higher venous injury rate [5–7]. Currently, all novel TLE techniques are used around the world depending on financial resources. Because of the diversity of TLE techniques and a relatively short time for their refinement, available reports on periprocedural risk are scarce and inconclusive.

## 2. Methods

We analyzed clinical data obtained from 1915 patients undergoing TLE at the Reference Center between 2006 and 2015. TLE was performed by a single operator, most frequently using different polypropylene Byrd dilator sheaths, rarely mechanical sheaths, tools for extraction via the femoral approach, or nonstandard tools for dissecting the proximal lead tips. Laser or electrosurgical dissection sheaths were not used.

### 2.1. Definitions

TLE was defined according to the 2017 HRS consensus guidelines [8] as any lead removal procedure in which at least one lead requires the assistance of equipment not typically required during implantation or at least one lead was implanted for longer than 1 year. Due to the necessity of using tools typical for TLE in the case of ICD leads implanted at least 0.5 years earlier, 35 (1.8%) of such extracted leads were included in the current analysis. A group of 35 patients with ICD leads implanted during > 0.5 years and below the 1 year consisted of patients who needed Byrd's dilators to remove the leads. The use of dilatators in these patients was obligatory due to the need to regain the venous access necessary to introduce further leads in noninfectious patients (30 cases). In patients with infections (5 cases) this was the access used to introduce the leads for temporary pacing, or access in case on various periprocedural complications.

Infectious and noninfectious indications for TLE were classified according to the 2017 HRS consensus guidelines. The efficacy of TLE was assessed also according to the 2017 HRS consensus guidelines as complete procedural success and clinical success including complete and partial radiographic success [8].

Complication of the procedure was regarded as permanently disabling if patients required emergency sternotomy or thoracotomy, developed significant deterioration of tricuspid valve (at least by 2 grades in echocardiography), and reported any, event mild, constant deterioration in the state of health.

Failure (procedural, clinical, and radiographic) referred to inability to achieve complete procedural or clinical success.

Major (MJC) and minor (MIC) complications were defined according to the 2017 HRS consensus guidelines [8].

Damage to the outer lead insulation in the intracardiac segment (15-20 cm from the lead tip), frequently exposing the conductor, detected during visual inspection was termed as intracardiac lead abrasion (ILA) [9].

### 2.2. Data Analysis

Factors potentially influencing the efficacy of TLE were analyzed in a group of 1915 patients in whom a total of 3207 leads had been explanted between 2006 and 2015.

Depending on complications occurrence the patients were divided into groups; first included uncomplicated patients (n=1790), second included patients with major complication (n=34) with subgroup of periprocedural death (n=7), and third included patients with minor complication only (n=91). In addition, a group of all patients who died during a 30-day follow-up was separated (n=28). We evaluated the effect of clinical and procedure-related factors such as type of the implanted device and procedures prior to TLE on the development of MJC and MIC as well as periprocedural and 30-day mortality. The data were collected and prepared for multivariate analysis to assess the factors that led someone to developing major (including periprocedural death) and minor complications during TLE.

Mortality at 30 days after TLE was evaluated on the basis of the data obtained from the Ministry of the Interior.

The study was approved by the local Bioethics Committee.

## 3. Statistical Analysis

Continuous variables were expressed as mean ± standard deviation and median with interquartile range (IQR). Categorical variables were reported as number and percentage. Due to large differences in the number of compared groups we decided to use nonparametric tests to compare differences between groups. Continuous variables were compared by Mann-Whitney U test, whereas categorical data were compared using the Chi-square test incorporating Yates correction.

### 3.1. Regression Analysis

Logistic regression analysis was performed to evaluate the relationship between the variables and the development of complications, whereas univariate and multivariate Cox proportional hazards regression model was used for analysis of 30-day survival. Multivariate logistic regression analysis with stepwise algorithm selection was performed to evaluate the relationship between the variables and the development of complications, whereas multivariate Cox proportional hazards regression model with stepwise algorithm selection was used for analysis of 30-day survival, and periprocedural deaths were excluded. The Kaplan-Meier curves of freedom from death were used to evaluate the effect of MJC and MIC on 30-day survival. Differences in survival data were compared with the log-rank test. Analogically to the Cox regression analysis, complete data and cut-off points were included in the log-rank test. Differences between groups were deemed statistically significant if the p value was <0.05 or when the 95% confidence interval did not include 1. Statistical analysis was performed with Statistica 10.0 software, Minneapolis, USA.

## 4. Results

Indications for TLE included cardiac device infections (CDI) in 773 (41.3%) patients and noninfectious indications (NI) in 1142 (58.7%) patients. A total of 3207 leads were extracted, including 2882 (89.9%) functional and 325 (10.1%) nonfunctional, abandoned leads. Complete procedural success was achieved in 94.78% of patients, clinical success in 97.86%, and complete and partial radiographic success in 94.78% and 94.83%, respectively. Periprocedural death occurred in 7 (0.4%) patients. There were 34 (1.8%) MJC and 91 (4.8%) MIC. In total, during 30-day follow-up 28 (1.46%) deaths occurred.

A baseline comparative analysis and univariate logistic regression analysis initially showed that the clinical factor significantly increasing the risk of MJC was female gender [OR: 3.345; p<0.001]. Increased risk for developing of MIC (30.2 % by 1 g/dl) was related to lower hemoglobin concentration [OR: 1.302; p<0,001]. The potential clinical factors significantly increasing the risk for developing MIC included female gender [OR: 3.354; p<0.01]; chronic renal failure (CRF) [OR: 2.189; p<0.05); and higher left ventricle ejection fraction (29.4% by 10%) [OR: 1.294; p<0.05]. The increased risk for MIC was additionally found in patients after sternotomy [OR:1.953; p<0.01], implantation of mechanical or bioprosthetic valves [OR: 2.464; p<0.01], and with lead-related infective endocarditis (LRIE) [OR: 1.777; p<0.01], especially isolated LRIE [OR: 2.155; p<0.01], i.e., without signs of pocket infection (PI) (Table 1).

From factors that were directly procedure-related, the risk for developing MJC (including periprocedural deaths) and MIC was significantly increased in patients with older leads, measured as dwell time of the oldest one extracted lead in patient: MJC [OR: 1.162; p<0.001] and MIC [OR: 1.069; p<0.001], mean dwell time of leads extracted in patient: MJC [OR: 1.196; p<0.001] and MIC [OR: 1.084; p<0.000], and sum of dwell time of leads extracted in single patient: MJC [OR: 1.073; p<0.001] and MIC [OR: 1.029; p<0.001]. The risk for developing MJC was also higher in patients undergoing atrial lead extraction [OR: 2.830; p<0.05] as well as simultaneous removal of leads from both sides of the chest [OR: 10.27; p<0.001]. Extraction of abandoned leads (and their number) was associated with more frequent occurrence of MJC, respectively: [OR: 2.110; p<0.001] and [OR: 2.427; p<0.001]. Removal of unipolar leads was associated with a significantly higher risk for developing MJC [OR: 3.166; p<0.001] and MIC [OR: 2.027; p<0.01]. A significant increase in the risk of MJC was also observed in patients undergoing extraction of more than four leads [OR: 4.093; p<0.01], via the right-side approach [OR: 5.008; p<0.01] or requiring a more complex approach to lead extraction [OR: 1.860; p<0.001]. Any technical problem during TLE also had a significant influence on the frequency of MJC [OR: 2.476; p<0.001], especially fibrotic adhesions binding the leads together which were associated with MJC occurrence [OR: 4.262; p<0.001] and periprocedural mortality [OR: 7.719; p<0.001) (Table 2).

Of the other procedure-related factors, the number of preceding procedures had a significant effect on developing both MJC [OR: 1.795; p<0.001] (including higher risk of periprocedural deaths [OR: 1.836; p<0.001]) and MIC [OR: 1.289; p<0.001]. The risk of complications increased significantly in relation with the number of abandoned leads (MIC [OR: 1.694; p<0.05]), (MJC [OR: 3.747; p<0.001]) and TLE of leads implanted on both sides of the chest (MJC [OR: 4.579; p<0.001]) (Table 2).

Potential device-related factors increasing the risk for developing MIC were sum of dwell times of leads in single patient before TLE [OR: 1.028; p<0.001], lead implantation under the age of 30 [OR: 2.234; p<0.005], and presence of intracardiac lead abrasions [OR: 2.680; p<0.001]. TLE of defibrillation leads was connected with decreased risk of MIC by 60% [OR: 0.400; p<0.01)] (Table 2, comparative analysis). Increased risk for developing of MIC was also related to lower hemoglobin concentration (32.4% by 1 g/dl) [OR: 1.324; p<0.001], lower BMI [OR: 1.064; p<0.05] (6.4% by 1 unit), and in absence of chronic antiplatelet therapy [OR: 2.083; p<0.01] (Table 1, comparative analysis).

A separate analysis demonstrated that the presence of vegetations (especially >2 cm) placed the patient at increased risk of periprocedural death and increased near five times the risk of death in 30-day follow-up [HR: 4.612; p<0.001] (Table 1, comparative analysis)). Risk of death in 30-day follow-up increased in patients with chronic renal failure [OR: 8.744; p<0.001] and diabetes [OR:2.172; p<0.05] too.

## 5. Multivariate Analysis

In view of the dominant influence of the system age on TLE complications, a separate analysis of the effect of each of the leads dwell time variants was made. For this purpose, three models of multivariate logistic regression were built based on the dwell time of the oldest one extracted lead in patient, mean dwell time of leads extracted in patient, and sum of dwell time of leads extracted in single patient.

The multivariate logistic regression after stepwise algorithm selection (p<0.2) showed that the strongest values in prediction of MJC are age of the extracted leads (increase in risk by 3.1 to 18.1% per year), female gender (increase in risk from 2.36 to 2.91 times), number of abandonment leads in patients (increase in risk by 65.3 to 75.7%), number of any technical problems during TLE (increase in risk by 36.9%), and number of previous CIED procedures in patient (increase in risk by 27.6%). Lower by 1g/dl of blood hemoglobin concentration was associated with an increase in the risk of MJC occurrence by 22.4% to 27.4% (Table 3).

Risk of MIC was higher in patients with previous sternotomy (increase in risk by 75.6% [OR: 1.756; p<0.05]), first implantation of CIED under the age of 30 (increase in risk 2.17 times [OR: 2.170; p<0.05]), malposition of the lead in the left ventricle (increase in risk 6.93 times [OR: 6.93; p<0.01]), lack of antiplatelet therapy (increase in risk by 72.4% [OR: 1.724; p<0.05]), and lower blood hemoglobin concentration (increase in risk by 33.2% per 1 g% of hemoglobin [OR: 1.332; p<0.001]) (Table 3).

**Figure 1:**
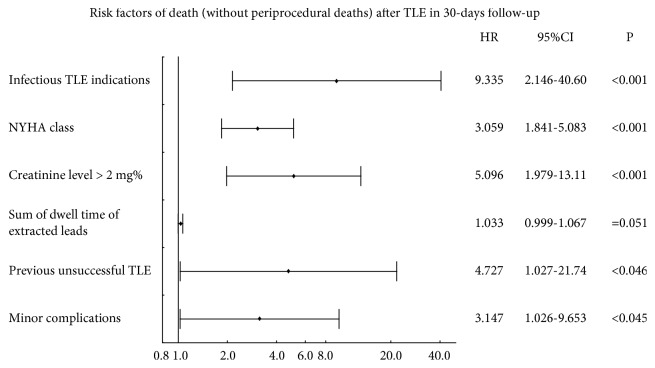
Risk factors of death in 30-day follow-up, results of multivariate stepwise Cox regression.

Multivariate Cox proportional hazards regression showed that the risk factors of death in 30-day follow-up were infective indications (increase risk over nine times [HR: 9.335; p<0.001]), a higher functional class of NYHA [HR: 3.059; p<0.001], chronic renal dysfunction [HR: 5.095; p<0.001], previous unsuccessful TLE [HR: 4.727; p<0.05], and MJC occurrence [HR: 3.147; p<0.05]. Sum of dwell time of leads extracted in single patient also worsened the prognosis but these parameters reached the borderline statistical significance [HR: 1.032; p=0.051] (Figure 1).

**Figure 2:**
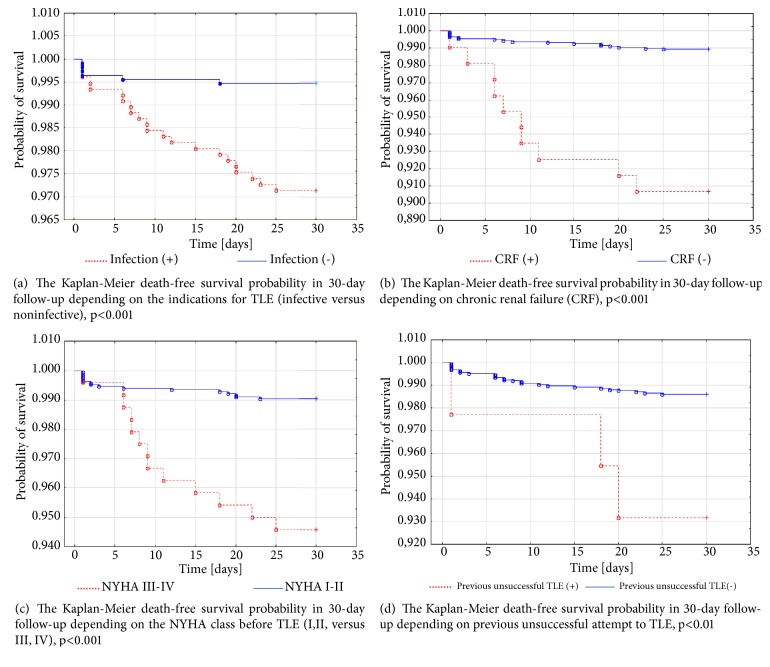
Kaplan–Meier death-free survival probability in 30-day follow-up depending on (a) failure of clinical success of TLE, (b) indications for TLE (infective versus noninfective), (c) NYHA class before TLE (I,II versus III, IV), and (d) previous unsuccessful attempt to TLE.

The Kaplan–Meier curves of time free from death depending on clinical success, NYHA class (I,II versus III,IV), infectious indications for TLE, and median of hemoglobin concentration in 30-day follow-up and results of log rang tests were presented in Figure 2.

## 6. Discussion

Transvenous lead extraction has become a common procedure worldwide. It is estimated that 10-15 thousand leads are removed annually and the need for lead extractions will probably continue to increase [10] in order to avoid life-threatening complications due to leaving not only the damaged but also potentially nonfunctional leads in place. In the present study, 58.7% of patients were submitted to TLE for noninfectious reasons. Probably in the world's largest TLE registry at the Cleveland Clinic 57.3% of patients underwent TLE for NI [11]. The findings of the present study demonstrated that multiple procedure-related factors such as lead implant duration, number and type of leads (especially atrial and unipolar leads), and number of preceding procedures involving the device (upgrading, implantation of additional leads) were the most important determinants of potential complications during TLE. Multivariate analysis demonstrated that because of increasing degrees of adhesion to myocardium and vascular walls, the time elapsed from initial lead insertion was the most significant predictor of MJC during TLE. Microscopic examination of the extracted leads confirmed that this process is time-dependent; the pathogenetic mechanism of lead adhesion is probably related to the inflammatory process, which is the foreign body response of the endomyocardium, accelerating fibrosis through inflow of inflammatory cells and in some cases also calcification [9]. Lead implant duration has already been identified as an important risk factor [3, 11–15]. In the present study, it was decidedly the most significant predictor of adverse outcomes. For this reason, multivariate analysis was performed taking into account different variables referring to lead implant duration (the sum of lead dwell times, average duration of the implanted lead, and implant duration of the oldest lead). The present analysis confirmed the significance of lead implant duration, with the sum of the individual dwell times of the extracted leads being probably the simplest factor to evaluate.

In the available literature, there is no detailed analysis of procedure-related factors. The world's largest registry of patients undergoing TLE at the Cleveland Clinic did not confirm the importance of procedure-related factors except the effect of lead implant duration on the development of major cardiovascular injury (MCVI) as well as the relationship between ICD lead removal and 30-day mortality in the univariate analysis. The multivariate analysis did not show the direct effect of the sum of lead dwell times, number, and type of extracted leads on the development of MJC [11]. Small observational studies provide contradictory results. Some investigators documented higher risk associated with extraction of biventricular, dual-coil defibrillator leads, and active fixation leads [8, 16, 17]. The reasons for this disparity, especially in comparison with the Cleveland registry, are not clear. However, it appears that relatively less attention has been paid to detailed analysis of procedure-related factors, focusing instead on clinical predictors, which exert a smaller effect on procedure-related risk but stronger on long-term survival. Additionally, the average duration of the implanted lead was shorter than that in the present study (61 versus 89 months). One should also bear in mind that power sheaths were used in 74.9% of procedures (laser sheaths in 70.1%) in Cleveland, which might have an overwhelming effect on the multivariate analysis. The study revealed that of all procedure-related factors powered sheaths were significantly associated with the development of major complications, especially MCVI [11].

The presence of abandoned, superfluous leads was another extremely important predictor of adverse patient outcomes associated with TLE in the present study. There is still much debate about whether we should leave abandoned leads in place or extract them. Although the Cleveland registry did not demonstrate increased risk associated with the presence of abandoned leads, recent reports from the same center evaluating patients undergoing TLE for infectious reasons confirmed apart from more frequent occurrence of vegetations, a higher rate of complications developing during the procedure [18]. According to the HRS guidelines extraction of superfluous functional and nonfunctional leads is a class 2b indication; i.e., it may be considered at the time of elective device replacement if contraindications are absent. In clinical practice decisions regarding extraction or leaving superfluous leads in place are made on a case-by-case basis. According to a single-center analysis comparing two management strategies, TLE was more frequently performed by experienced, high-volume operators. The study demonstrated also that the course of the procedure and the presence of MJC and MIC as well as 3-year mortality were comparable in patients with and without superfluous leads. However, the study was performed in a small group of patients in whom a total of 488 procedures were evaluated [19]. The present investigation, apart from showing a significantly higher risk of TLE in patients with abandoned leads, confirmed also that every year that elapsed from primary implantation increased the risk by 16%. For this reason, abandonment of superfluous leads is a strong predictor of adverse outcomes. Additionally, the presence of the foreign body itself is a risk factor for developing infectious complications [20]. The concerns about removing leads that perforate myocardial walls appear no longer valid because the efficacy of the procedure remains unchanged in such cases. Moreover, abandonment of leads means “overperformance” of procedures such as upgrading or placement of a new lead. However, the present findings reveal that every procedure prior to transvenous lead extraction significantly increases the risk associated with lead removal.

Of the clinical factors in the present study female sex and low hemoglobin levels were the most important predictors of adverse outcomes following TLE. Although in previous investigations women were found to have more complications [8, 16], the causes of the higher complication rate are not clear. Men with CIED probably more often develop infectious complications, and TLE is performed earlier than in women, meaning that leads with shorter implant durations are extracted. In women one of the reasons may be increased fragility of the vessels making them prone to damage. Anemia was another clinical predictor of adverse outcomes after TLE. Poorer outcomes in patients with anemia undergoing cardiac and noncardiac surgery have already been reported but not fully explained. An increased incidence of red cell transfusion in the perioperative period and multiple chronic conditions or a high probability of undiagnosed generalized disease in such patients are usually put forward as a possible cause [21, 22]. As for TLE, a negative effect of anemia was observed in the Cleveland Clinic registry combined with an elevated INR as a predictor of 30-day mortality [11]. In the present study, low hemoglobin levels had an impact on the immediate results, increasing the risk of the procedure itself. Probably low hemoglobin levels are associated with vascular fragility and hypoxia of vascular walls, making them more prone to disruption during catheter manipulation.

In the present study, apart from evaluating predictors of major complications, we analyzed also minor complications, which appeared to influence significantly 30-day mortality after TLE. Risk factors for MIC were similar to those observed for major ones; however, in the multivariate analysis, a significant effect of the implantation of the system at the young age and the history of sternotomy was demonstrated. A potentially higher procedural risk in patients with implanted devices younger than 30 years is especially worth emphasizing, because there is still a belief in the high risk of TLE in older people. It is however noteworthy that none of the available studies documented the effect of patient older age on the procedure efficacy. Moreover, previous investigators demonstrated the high efficacy of TLE in patients aged 80 and 90 years, comparable to that in younger subjects [23].

The present study describes mainly procedure-related predictors of adverse outcomes in patients undergoing TLE; however evaluation of survival is very important. As shown in the present study 30-day mortality was affected by entirely different determinants, mainly the clinical ones such as heart failure, renal failure, and device infections. Most of these factors were demonstrated in the Cleveland registry [11]. However, in the present study, the very important impact of successfully and safety performed TLE on short-term survival after procedure was demonstrated. Knowledge of TLE risk factors should contribute to the application of appropriate precautions during the most difficult procedures.

## 7. Study Limitations

Multivariate analysis of such a large number of procedure-related factors is difficult to perform because of mutually exclusive determinants and marked predominance of one parameter over the other. In this situation, trivariate analysis was used to evaluate most reliably the effect of individual factors on the risk associated with TLE.

## 8. Conclusions

Procedure-related variables have been found to be the most important predictors of adverse outcomes in patients undergoing transvenous lead extraction. A better understanding of these determinants allows for implementing strategies that minimize procedure-related risk and improve the efficacy and safety of TLE. Female sex and anemia were found to be the only significant clinical, patient-dependent factors. It is noteworthy that other clinical factors, frequently analyzed by other investigators such as heart failure, chronic renal failure, and pacemaker infections did not determine the immediate results of transvenous lead extraction. These factors together with minor complications influenced 30-day mortality after TLE and for this reason should be monitored closely in the periprocedural period.

## Figures and Tables

**Table 1:** Clinical risk factor analysis of transvenous lead extraction.

Variables	All patients	Uncomplicated procedures	Major complications	Minor complications	Periprocedural death	30-day mortality
	all procedures					
Number of patients (number, %)	1915 (100%)	1790 (93.5%)	34 (1.8%)	91 (4.8%)	7 (0.4%)	28 (1.5%)

**Patient-dependent risk factors**						

Patient's age at TLE (mean, SD median, IQR)	64.83	64.99	63.68^NS^	61.74^NS^	70.70^NS^	71.50^*∗*^
	±15.95	±15.73	±17.20	±18.91	±11.06	±10.20
	68.00	68.00	67.00	69.00	72.00	72,00
	[58.00-76.00]	[58.00-76.00]	[53-76]	[56.00-76.00]	[65.00-80.00]	[65.00-80.00]

Female gender (n, %)	822 (42.90)	749 (41.20)	24^*∗∗*^ (70.60)	49^*∗*^ (53.80)	5^NS^ (71.4)	16^NS^ (57.1)

BMI (kg/m^2^) (mean, SD median, IQR)	27.48	27.29	26.94^NS^	26.39^*∗*^	26.12^NS^	26.75^NS^
	±6.82	±4.12	±3.59	±3.96	±1.86	±4.68
	27.14	27.28	26.70	25.83	26.37	25.76
	[24.03-30.44)	[24.03-30.47]	[24.86-30.19]	[22.49-29.75]	[25.48-27.24]	[21.23-27.76]

NYHA functional class (mean, SD median, IQR)	1.64	1.64	1.50^NS^	1.59^NS^	1.57^NS^	2.14^*∗∗∗*^
	±0.72	±0.72	±0.71	±0.71	±0.79	±0.89
	2 [1-2]	2 [1-2]	1 [1-2]	1[1-2]	1[1-2]	2[1-3]

NYHA classes I and II (n, %)	1675 (87.50)	1566 (87.50)	30^NS^ (88.20)	79^NS^ (86.80)	6^NS^ (85.7)	17^*∗∗∗*^ (60.7)

NYHA classes III and IV (n, %)	240 (12.50)	224 (12.50)	4^NS^ (11.80)	12^NS^ (13.20)	1^NS^ (14.3)	11^NS^ (39.3)

Left ventricular ejection fraction LVEF (mean, SD median, IQR)	38.91	38.74	41.22^NS^	40.98^NS^	38.57^NS^	31.74^*∗∗*^
	±13.40	±13.40	±13.26	±13.48	±14.64	±14.03
	41.88	41.88	45.54	44.53	42.00	34.95
	[30.24-46.53]	[29.47-46.53]	[34.01-48.59]	[37.37-47.71]	[29.50-50.00]	[21.28-37.99]

Diabetes types I and II (n, %)	357 (18.6)	335 (18.7)	4^NS^ (11.8)	18^NS^ (19.8)	1^NS^ (14.3)	10^*∗*^ (35.7)

Chronic renal failure (CRF) creatinine level (mean, SD median, IQR)	1.17	1.16	1.19^NS^	1.46^NS^	1.29^NS^	2.06^*∗∗∗*^
	±0.73	±0.69	±0.67	±1.30	±0.62	±1.61
	1.00	1.00	1.00	1.10	1.10	1.50
	[0.84-1.28]	[0.88-1.25]	[0.80-1.25]	[0.80-1.30]	[1.00-1.30]	[1.20-2.10]

CRF creatinine level>2 mg/dl (n, %)	107 (5,59)	93 (5.20)	4^NS^ (11.8)	10^*∗*^ (11.0)	1^NS^ (14.3)	10^*∗∗∗*^ (35.7)

Malignancy (n, %)	116 (6.06)	109 (6.09)	2^NS^ (5.88)	5^NS^ (5.49)	1^NS^ (14.3)	2^NS^ (7.14)

Hemoglobin concentration (mean, SD median, IQR)	13.17	13.2	11.97^*∗∗*^	12.09^*∗∗∗*^	12.60^NS^	11.49^*∗∗∗*^
	±2.79	±1.83	±2.23	±2.29	±2.00	±2.59
	13.30	13.40	12.15	11.80	13.00	12.20
	[12.00-14.40]	[12.10-14.40]	[10.40-14.00]	[10.70-13.70]	[11.50-14.20]	[8.80-13.60]

Hemoglobin concentration<12 g/dl (n,%)	463 (24.18)	396 (22.12)	15^*∗∗*^ (44.12)	43^*∗∗∗*^ (47.25)	2^NS^ (28.57)	14^*∗∗*^ (50)

Valvular cardiac implant (n, %)	113 (5.90)	100 (5.59)	1^NS^ (2.94)	12^*∗∗*^ (13.3)	0^NS^ (0.00)	3^NS^ (10.7)

Prior sternotomy (n, %)	291 (15.2)	256 (14.90)	2^NS^ (5.88)	23^*∗*^ (25.3)	0^NS^ (0.00)	8^NS^ (28.6)

Permanent AF (n, %)	429 (22.4)	406 (22.7)	5^NS^ (14.7)	18^NS^ (19.8)	2^NS^ (28.6)	10^NS^ (35.7)

Chronic anticoagulation (n, %)	653 (34.1)	617 (34.5)	8^NS^ (23.5)	28^NS^ (30.8)	2^NS^ (28.6)	11^NS^ (39.3)

Chronic antiplatelet therapy (n, %)	798 (41.7)	764 (42.7)	10^NS^ (29.4)	24^*∗*^ (26.4)	1^NS^ (14.3)	10^NS^ (35.7)

Cardiac device infections (n, %)	773 (41.3)	716 (40.0)	12^NS^ (35.3)	45^NS^ (49.5)	5^NS^ (71.4)	22^*∗∗∗*^ (78.6)

Lead-related infective endocarditis (LRIE) ( n%)	527 (27.5)	480 (26.8)	11^NS^ (32.4)	36^*∗*^ (39.6)	4^NS^ (57.1)	19^*∗∗∗*^ (67.9)

Isolated LRIE (n, %)	217 (11.3)	191 (10.7)	7^NS^ (20.6)	19^*∗∗*^ (20.9)	3^NS^ (42.9)	11^*∗∗∗*^ (39.3)

Pocket infection (PI) (n, %)	556 (29.0)	525 (29.3)	5^NS^ (14.7)	26^NS^ (28.6)	2^NS^ (28.6)	11^NS^ (39.3)

Vegetations (n, %)	383 (20.0)	347 (19.4)	7^NS^ (20.6)	29^NS^ (31.9)	2^NS^ (28.6)	14^*∗∗∗*^ (50.0)

Large vegetations (2-4 vs. 0-1 cm) (n, %)	209 (10.9)	182 (10.2)	6^NS^ (17.6)	21^NS^ (23.1)	2^*∗∗*^ (28.6)	9^*∗∗*^ (32.1)

Vegetations connected with heart wall (n, %)	37 (1.93)	34 (1.90)	0^NS^ (0.00)	4^NS^ (4.40)	0^NS^ (0.00)	2^NS^ (7.14)

Multiple vegetations (n, %)	191 (9.97)	173 (9.66)	3^NS^ (8.82)	15^NS^ (16.5)	1^NS^ (14.3)	7^*∗*^ (25.0)

NS: nonsignificant; *∗*P < 0.05; *∗∗*P < 0.01; *∗∗∗*P < 0.001 (when compared to noncomplicated procedures).

**Table 2:** Procedure-related risk factor analysis of transvenous lead extraction.

Potential risk factors	All patients	Uncomplicated procedures	Major	Minor complications	Periprocedural death	30-day mortality
	all procedures		complications			
Number of patients (n, %)	1915 (100%)	1790 (93.5%)	34 (1.8%)	91 (4.8%)	7 (0.4%)	28 (1.5%)

**Device-related risk factors**						

Number of active leads in system (mean, SD median, IQR)	1.90	1.81	1.76^NS^	1.76^NS^	2.00^NS^	1.71^NS^
	±0.64	±0.64	±0.61	±0.60	±0.82	±0.71
	2[1-2]	2[1-2]	2[1-2]	2[1-2]	2[1-2]	2[1-2]

Presence of defibrillation lead -ICD (VVI or DDD) (n, %)	513 (26.80)	496 (27.10)	4^NS^ (11.80)	13^*∗*^ (14.30)	2^NS^ (28.6)	4^NS^ (14.3)

CRT-D system (n, %)	106 (5.53)	101 (5.64)	1^NS^ (2.94)	4^NS^ (4.40)	1^NS^ (14.3)	1^NS^ (3.57)

CRT-P system (n, %)	54 (2.82)	52 (2.91)	0^NS^ (0.0)	2^NS^ (2.20)	0^NS^ (0.00)	1^NS^ (3.57)

Lead/system implanted under age of 30 (n, %)	173 (9.03%)	149 (8.32%)	8^*∗∗*^ (23.5%)	16^*∗∗*^ (17.6%)	0 (0.00%)	1 3.57%

Sum of dwell times of all leads in cardiovascular system (CVS) before TLE (years) (mean, SD median, IQR)	13.75	13.08	33.29^*∗∗∗*^	18.48^*∗∗∗*^	30.37^*∗∗*^	19.24^NS^
	±11.95	±11.15	±20.52	±12.31	±20.0	±16.57
	10.33	9.92	30.00	15.67	29.17	13.50
	[5.17-18.50]	[5.00-18.00]	[20.00-38.17]	[9.42-27.08]	[9.25-41.25]	[7.33-28-00]

Intracardiac lead abrasion (ILA) (n, %)	423 (22.10)	473 (20.80)	12^NS^ (35.30)	38^*∗*^ (41.8)	2^NS^ (28.6)	0^*∗*^ (0.00)

**Previous procedure-related risk facto**rs						

Number of CIED-related procedures before TLE (mean, SD median, IQR)	1.92	1.89	3.29^*∗∗∗*^	2.27^*∗∗∗*^	3.57^*∗∗*^	2.54^*∗*^
	±2.60	±2.62	±1.78	±1.24	±2.15	±1.60
	2 [1-2]	2[1-2]	3 [2-4]	2 [1-3]	2 [2-6]	2 [1-3]

Presence of abandoned lead(s) before TLE (n, %)	280 (14.6)	247 (13.80)	13^*∗∗*^ (38.2)	20^*∗*^ (22.0)	3^NS^ (42.9)	7^NS^ (25.0)

Number of abandoned leads per patient (mean, SD median, IQR)	0.20	0.19	0.50^*∗*^	0.26^*∗*^	0.71^NS^	0.43^NS^
	±0.55	±0.52	±0.86	±0.61	±1.25	±0.84
	0	0	0	0	0	0
	[0-0]	[0-0]	[0-2]	[0-0]	[0-2]	[0-0]

Leads on both sides of the chest (n, %)	90 (4.70)	79 (4.41)	6^*∗∗*^ (17.6)	5^NS^ (5.49)	1^NS^ (14.3)	2^NS^ (7.14)

Previous upgrade with lead abandonment (n, %)	121 (6.32)	105 (5.87)	5^NS^ (14.7)	11^*∗*^ (12.1)	2^NS^ (28.6)	6^*∗∗*^ (21.4)

Lead-related venous occlusion or severe stenosis (n, %)	632 (33.00)	586 (32.70)	16^NS^ (47.1)	30^NS^ (33.0)	4^NS^ (57.1)	9^NS^ (32.1)

Previous unsuccessful attempt of lead removal (n, %)	44 (2.30)	38 (2.12)	2^NS^ (5.88)	4^NS^ (4.40)	1^NS^ (14.3)	3^*∗*^ (10.7)

**TLE procedure-related risk factors**						

Dwell time of the oldest extracted lead per patient (years) (mean, SD, median, IQR)	7.61	7.31	16.35^*∗∗∗*^	10.38^*∗∗∗*^	16.35^*∗∗∗*^	14.73^*∗∗*^
	±5.87	±5.59	±9.00	±6.54	±9.00	±10.85
	7.61	6.00	15.25	9.42	13.33	9.25
	[3.25-10.42]	[3.16-10.08]	[10.17-20.42]	[5.50-14.25]	[8.33-19.33]	[4.83-14.58]

Average dwell time of leads extracted per patient (years) (mean, SD median, IQR)	6.87	6.63	13.55^*∗∗∗*^	9.22^*∗∗∗*^	13.55^*∗∗∗*^	12.12^*∗∗*^
	±4.98	±4.81	±6.75	±5.18	±6.75	±6.75
	5.83	5.60	13.45	8.83	10.32	8.08
	[3.17-9.25]	[3.00-9.08]	[9.04-19.08]	[5.00-13.00]	[8.83-19.33]	[3.67-14,00]

Sum of dwell time of extracted leads in patient (years) (mean, SD median, IQR)	12.02	11.44	29.59^*∗∗∗*^	16.76^*∗∗∗*^	33.29^*∗∗∗*^	30.37^*∗∗*^
	±11.20	±10.55	±20.72	±12.40	±20.52	±20.0
	8,50	8.17	27.25	13.00	27.25	13.50
	[4.08-16.50]	[4.00-15.83]	[16.42-36.33]	[7.00-24.83]	[9.25-29.17]	97.33-27.25]

HV/ICD lead extraction (n, %)	482 (25.2)	467 (26.1)	4^NS^ (11.8)	11^*∗*^ (12.1)	2^NS^ (28.6)	4^NS^ (14.3)

Coronary sinus (CS) lead extraction (n, %)	262 (13.7)	248 (13.9)	5^NS^ (14.7)	9^NS^ (9.98)	3^NS^ (42.9)	5^NS^ (17.9)

Right atrial (RA) lead extraction (n, %)	1128 (58.9)	1048 (58.5)	27^*∗*^ (79.4)	53^NS^ (58.2)	5^NS^ (71.4)	18^NS^ (64.3)

Right ventricular (RV) lead extraction (n, %)	1650 (86.2)	1540 (86.0)	28^NS^ (82.4)	82^NS^ (90.1)	5^NS^ (71.4)	24^NS^ (85.7)

Extraction of leads implanted on both sides of the chest during the same TLE procedure (n, %)	35 (1.83)	27 (1.51)	5^*∗∗∗*^ (14.7)	3^NS^ (3.30)	1^NS^ (14.3)	1^NS^ (3.57)

Extraction of abandoned lead (n, %)	237 (12.4)	216 (12.1)	10^*∗∗*^ (29.4)	11^NS^ (12.1)	0^NS^ (0.00)	7^NS^ (25.0)

	0.17	0.16	0.50^*∗*^	0.26^*∗*^	0.29^NS^	0.39^*∗*^
Number of extracted abandoned leads per patient (mean, SD median, IQR)	±0.50	±0.48	±0.86	±0.61	±0.76	±0.79
	0	0	0	0	0	0
	[0-0]	[0-0]	[0-1]	[0-0]	[0-0]	[0-0]

Unipolar (UP) atrial (A) lead extraction (n, %)	102	86 (4.80)	7^*∗∗∗*^ (20.6)	9^NS^ (9.89)	7^*∗∗∗*^ (100.0)	0^NS^ (0.00)

UP ventricular (V) (PM) lead extraction (n, %)	184	161 (8.99)	8^*∗*^ (23.5)	15^*∗*^ (16.5)	0^NS^ (0.00)	0^NS^ (0.00)

UP A+V (PM) lead extraction (n, %)	229	195 (10.9)	15^*∗∗*^ (44.1)	19^*∗*^ (20.9)	15^*∗∗*^ (44.1)	0^NS^ (0.00)

Extraction of UP leads above median age (n, %)	137	106 (5.92)	15^*∗∗∗*^ (44.1)	16^*∗*^ (17.6)	0^NS^ (0.00)	2^NS^ (7.14)

Extraction of 4 and more leads during TLE procedure (n, %)	81 (4.23)	71 (4.00)	5^*∗∗*^ (14.7)	5^NS^ (5.50)	1^NS^ (14.3)	1^NS^ (3.57)

Any technical problems during TLE (n, %)	294 (15.3)	256 (14.3)	13^*∗∗∗*^ (38.2)	25^NS^ (27.5)	3^NS^ (42.9)	6^NS^ (21.4)

NS: nonsignificant; *∗*P < 0.05; *∗∗*P < 0.01; *∗∗∗*P < 0.001 (when compared to noncomplicated procedures).

**Table 3:** Risk factors of minor and major complications—multivariate logistic stepwise regression.

	**HR**	**95**%**CI**	**p**
**Major complications: multivariate regression based on dwell time of the oldest one extracted lead in patient**			

Dwell time of the oldest one [1 year]	1.138	1.090-1.188	0.000

Female gender	2.362	1.098-5.078	0.028

Hemoglobin concentration [1g/dl]	1.271	1.064-1.515	0.008

Number of abandoned leads in the patient (by one)	1.653	1.086-2.515	0.019

**Major complications: multivariate regression based on mean dwell time of leads extracted in patient**			

Mean dwell time of leads [1 year]	1.181	1.118-1.247	0.000

Female gender	2.390	1.103-5.180	0.027

Hemoglobin concentration [1g/dl]	1.274	1.066-1.524	0.008

Number of any technical problems during TLE	1.369	1.017-1.841	0.038

Number of abandoned leads in the patient (by one)	1.757	1.126-2.742	0.013

**Major complications: multivariate regression based on sum of dwell time of leads extracted in single patient**			

Sum of dwell time of leads (1 year)	1.056	1.031-1.082	0.000

Female gender	2.709	1.266-5.797	0.010

Hemoglobin concentration [1g/dl]	1.224	1.020-1.468	0.029

Number of previous procedures in patient (by one)	1.276	0.980-1.662	0.070

**Minor complications**			

Hemoglobin concentration [1g/dl]	1.332	1.198-1.479	0.000

Previous sternotomy	1.756	1.062-2.905	0.028

Lack of antiplatelet therapy	1.724	1.052-2.825	0.031

First implantation of CIED under the age of 30	2.170	1.195-3.940	0.011

Malposition of the lead in the left ventricle	6.930	1.360-35.318	0.020

## Data Availability

The data from the TLE database maintained by the main operator used to support the findings of this study are included within the article.
